# Hydrogels loaded with different substances for treating heart failure: a promising therapy

**DOI:** 10.3389/fcvm.2026.1744301

**Published:** 2026-06-03

**Authors:** Ran Meng, Weiqiang Xiao, Shisen Liang, Jiahui Shen, Hanlin Xu, Haojun Li, Xiaohong Xue, Mingqi Zheng, Xugang Wang, Mei Wei

**Affiliations:** 1 Department of Heart Center, the First Hospital of Hebei Medical University, Shijiazhuang, Hebei, China; 2 Graduate School of Hebei Medical University, Shijiazhuang, Hebei, China; 3 Hebei Medical University, Shijiazhuang, Hebei, China; 4 Department of Cardiology, Xingtai Renze District People’s Hospital, Xingtai City, Hebei, China

**Keywords:** AMI, biomedical materials, function, hydrogels, myocardial

## Abstract

Acute myocardial infarction (AMI), which causes cardiomyocyte death due to ischemia, affects ∼7 million people annually worldwide, and its mortality rate is greater than one-third. Furthermore, AMI severely impairs cardiac function, leading to heart failure and fatal arrhythmias. While timely reperfusion improves survival, adverse remodeling and subsequent heart failure remain major challenges. After cardiomyocyte death, excessive fibrosis in and around the infarct decreases heart size and impairs cardiac function, leading to heart failure. Current end-stage treatments (e.g., drugs and devices) cannot repair damaged tissue, but injectable biomaterials, particularly hydrogels, offer promising new therapeutic strategies; their excellent biocompatibility, degradability, high water content, and injectability allow the targeted delivery of bioactive molecules, drugs, cells, and exosomes (exos) directly into the damaged myocardium to promote repair after AMI. Here, we describe the mechanism of progression from coronary atherosclerotic heart disease to myocardial infarction and myocardial injury and the superiority of hydrogels for treating this disease. We also discuss the mechanisms of action of different bioactive molecules, drugs, cells, exos, miRNA and two therapeutic agents-loaded hydrogels for treating myocardial injury and the experimental effects of these hydrogels. Finally, we discuss the existing problems associated with injectable hydrogels and the prospects of using hydrogel formulations in the treatment of myocardial infarction.

## 1 Background

Acute myocardial infarction (AMI) is the progressive deterioration of the pumping function of the heart due to ischemia-induced cardiomyocyte death. The cardiomyocyte death can occur within minutes of ischemia (1). During the acute phase, the necrotic myocardium progressively shrinks, resulting in thinning of the ventricular wall and decreased mechanical support. Scar formation in this region helps prevent aneurysm rupture and further deterioration of cardiac function. Although such adaptive remodeling is critical for preventing early death in patients with AMI, excessive and persistent fibrosis in the infarct area and surrounding areas can significantly affect ventricular muscle size and function, ultimately leading to heart failure (2). AMI affects approximately 7 million people worldwide each year and is associated with a mortality rate of more than one-third, making it a major threat to life and health (3). Early and timely reperfusion therapy significantly improves the survival of patients with AMI (4). Nevertheless, the resulting adverse remodeling and subsequent episodes of heart failure remain major clinical challenges (5). The high morbidity, mortality and rehospitalization rates of AMI-induced heart failure place a great economic and psychological burden on patients. Current treatments for end-stage heart failure after AMI include drug therapy and left ventricular assist device implantation, but these therapies have difficulty repairing the damaged myocardium (6). Ultimately, this lack of repair leads to the deterioration of pump function; furthermore, left ventricular assist devices are expensive, making them unsuitable for all heart failure patients. Heart transplantation is the only effective treatment for end-stage heart failure, but its efficacy is limited by factors such as the scarcity of donors and immune rejection after transplantation. In addition, the necessary long-term use of immunosuppressants may increase the risk of infection and malignancy (7). Therefore, there is an urgent need for new therapies for managing AMI.

Regenerative medicine is an emerging science with the potential to regenerate or repair damaged cardiac tissues, and it is a promising approach for addressing this global challenge (8). Hydrogels are promising candidate materials in regenerative medicine; consisting of hydrophilic polymer networks, they possess excellent biocompatibility, degradability, and high water solubility, are easy to inject, and can simulate the specific physical characteristics of the tissue microenvironment and extracellular matrix (ECM) (9, 10). And the physicochemical properties of these hydrogels can be finely tuned to match the specific needs of the damaged myocardium. Parameters such as gelation rate, mechanical strength, and degradation kinetics can be optimized to support tissue regeneration while minimizing potential side effects. Some hydrogels are designed to respond to the local environment, releasing therapeutic payloads in response to pH changes or enzymatic activity, which further enhances their effectiveness (11, 12).

In addition, injectable hydrogels offer a versatile platform for delivering a wide range of therapeutic agents, including growth factors, stem cells, and drugs. This capability enables a synergistic approach to cardiac repair, where multiple therapeutic effects are combined within a single treatment (13). For example, some hydrogels can simultaneously promote angiogenesis, modulate the inflammatory response, and enhance the proliferation of cardiac progenitor cells ((CPCs)-which can replenish adult injured cardiomyocytes and vascular cells through differentiation) (14–16). This multi-modal functionality increases the overall efficacy of the intervention and addresses the complex pathophysiology of myocardial injury more comprehensively than single-agent therapies. Overall, injectable hydrogels represent a versatile and powerful strategy for myocardial repair, offering the unique advantage of combining multiple therapeutic mechanisms to promote more complete and lasting cardiac recovery.

In this article, we describe the mechanism of progression from coronary atherosclerotic heart disease to myocardial infarction and myocardial injury; the superiority of hydrogels for treating this disease; the mechanisms of action of different bioactive molecules, drugs, cells, exosomes (exos), and miRNA-loaded hydrogels for treating myocardial injury; and the experimental effects of these hydrogels in recent five years to optimize treatment options. Finally but equally importantly, we discuss the existing problems associated with injectable hydrogels and the prospects of using hydrogel formulations in the treatment of myocardial infarction.

## 2 Pathological mechanisms of AMI and myocardial damage after AMI

**Figure 1:**
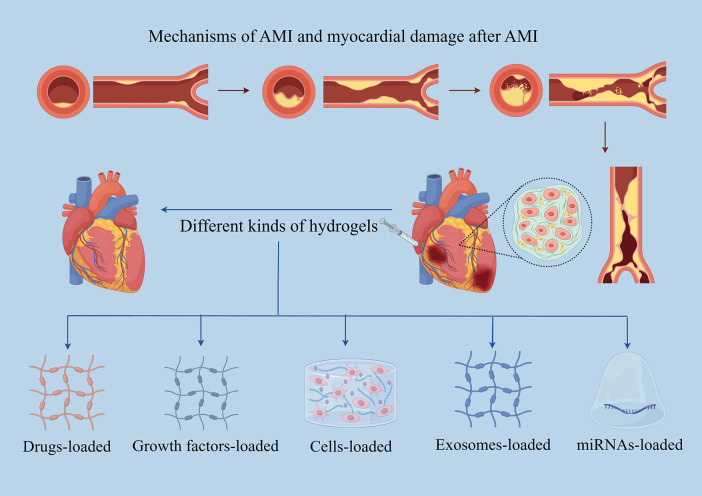
Pathological mechanisms of AMI and myocardial damage after AMI. The development of atherosclerosis goes through three stages: (a) the initial lipid streak stage, (b) the fibrous plaque stage, and (c) the advanced lesion and thrombosis stage. As the plaque progresses, the coronary artery lumen gradually narrows, eventually leading to a reduction or interruption of blood flow to a certain part of the heart. This reduced blood flow promotes the formation of coronary artery occlusive blood clots, thereby causing myocardial ischemia and infarction. After AMI, the hypoxia and insufficient blood supply of the damaged myocardial tissue lead to the death/apoptosis of myocardial cells, the release of pro-inflammatory subcellular components and other damage signals, and the stimulation of ECM degradation. Hydrogel is a material with a unique three-dimensional cross-linked polymer network composed of natural or synthetic hydrophilic polymers with many chemical components. Usually, hydrogels are designed as injectable liquid solutions that will gel into a three-dimensional polymer network upon reaching the target area and quickly combine with the surrounding tissues. Hydrogels can increase the thickness of the damaged myocardial tissue, support the ventricular wall, delay left ventricular remodeling, promote cell adhesion, reduce scar tissue, and also act as ECM-like substances, continuously delivering local biological factors, accelerating angiogenesis, and ultimately achieving improved cardiac function.

AMI can be divided into several types: type 1 is associated with atherosclerosis; type 2 is due to insufficient oxygen supply to cardiomyocytes; type 3 is defined as sudden cardiac death; and types 4 and 5 are associated with procedures such as percutaneous coronary intervention and coronary artery bypass grafting, respectively (1). Of these, type 1—the type associated with atherosclerosis—is the most common cause of AMI. The development of atherosclerosis can be divided into three distinct stages: (a) the initial lipid streak stage, (b) the fibrous plaque stage, and (c) the late lesion and thrombosis stage (17). During the lipid streak stage, various forms of lipids are retained and accumulate in the arterial wall. Macrophages infiltrate the vascular intima, absorb excess lipids and form foam cells. In later stages, vascular smooth muscle cells migrate into the innermost layer of the artery and form fibrous caps in areas of atherosclerosis (18). This thick fibrous cap contributes to the stability of the plaque. However, excessive foam cell accumulation can lead to plaque destabilization and rupture (19) that bring the blood into contact with the subendothelial matrix and plaque contents, which interact with the clotting factors and cells present in the blood, triggering thrombosis (20). All types of AMI is caused mainly by a reduction in or interruption of blood flow to a part of the heart. This reduction in blood flow promotes the formation of occlusive blood clots in the coronary arteries, which can trigger myocardial ischemia and infarction (21) (Figure 1).

The pathological changes following AMI comprise a process involving three phases: inflammation, proliferation and remodeling (22). The inflammatory phase is the early phase in which inadequate oxygen and blood supply to myocardial tissue leads to cardiomyocyte death/apoptosis, the release of proinflammatory subcellular components and other damage signals, the stimulation of ECM degradation and the alteration of normal cell function (23). Damaged cells upregulate nuclear factor-kappa β (NF-κβ)-dependent cytokines and chemokines and recruit many neutrophils that infiltrate the infarct site. In response to the specific myocardial environment, monocytes differentiate into macrophages that engulf dead cells and ECM debris and produce growth factors (e.g., basic fibroblast growth factor (bFGF) and vascular endothelial growth factor (VEGF)) that activate angiogenesis (24). During the progressive proliferative phase, the suppression and resolution of inflammation are accompanied by the migration of many fibroblasts into the damaged area, where they transform into myofibroblasts and begin to synthesize large amounts of ECM for repair. After a few weeks, collagen fibers form throughout the infarct area, replacing the necrotic cells. Eventually, the collagen deposits form scars, and the infarct area rebuilds (25).

Hydrogels are materials with a unique three-dimensional cross-linked polymer network composed of natural or synthetic hydrophilic polymers with numerous chemical components. Usually, hydrogels are designed for injection as a liquid solution, which will gel into a three-dimensional polymer network upon reaching the target area, quickly integrating with the surrounding tissues. Because the heart is constantly in a contraction-expansion movement state, hydrogels become easily transferable in a liquid state but remain gel-like after gelation to increase the cell survival rate and/or biological molecule activity (16). As an implant material for myocardial infarction treatment, hydrogels have the following advantages: (1) they can increase the thickness of damaged myocardial tissue, support the ventricular wall, delay left ventricular remodeling, promote cell adhesion, reduce scar tissue and improve cardiac function; (2) they can function as ECM-like substances, sustainably delivering biological factors locally and accelerating angiogenesis; and (3) as they can be delivered via injection methods, the trauma and surgical complications experienced by the patients are reduced, as the rapid effect of the injection transfers the load on adjacent muscle cells to the hydrogel (26).

## 3 Medication-loaded hydrogels

The current guidelines clearly state that drugs are used to treat heart failure. Systemic administration via oral or intravenous routes may cause side effects of the drugs. Therefore, we describe the effect of delivering drugs through hydrogel delivery systems (Table 1).

**Table 1:** Medications-loaded into different hydrogels, the signaling pathways/cytokines that their administration involves, and the corresponding effects.

Hydrogels	Animal model	The time of delivery	Signaling pathways/cytokines	The duration of treatment	Effects	References
PEG-PBA/HA-SH/AST NPs/GNRs	rats	–	TAK1-MAPK, TLR4/NF-κB	28days	1. Inhibit apoptosis. 2. Promote angiogenesis. 3. Promote electrical signaling in the myocardium. 4. Delay ventricular remodeling and reverse impaired cardiac function.	(27)
Triptolide/PLGA/Pluronic F127	rats	–	NF-κB	28 days	1. Inhibit apoptosis. 2. Prevent ventricular dilation and wall thinning. 3. Improve cardiac function. 4. Inhibit myocardial fibrosis.	(28)
Salvianolic acid B/ HA-NB/WPI-MA hydrogel	mice	After LAD ligation	PARP-1	28 days	1. Scavenge excessive ROS. 2. Inhibit the inflammatory response. 3. Relive overactivation of the RAAS.	(29)
EGCG/Rhein hydrogel	mice	5 mintes before loosening LAD	TLR4/NF-κB, AKT/GSK3β, AMPK/FGF23	28 days	1. Scavenge ROS. 2. Reduce cardiomyocyte apoptosis. 3. Improve cardiomyocyte survival. 4. Restore cardiac function and reduce fibrosis.	(30)

PEG-PBA, polyethylene glycol diacrylate/4-vinyl phenylboronic acid; HA-SH, thiol hyaluronic acid; GNRs, gold nanorods; AST NPs, astragaloside IV nanoparticles; TAK1-MAPK, transforming growth factor-β-activated kinase 1 mitogen-activated protein kinase; TLR4, toll-like receptor 4; NF-κB, nuclear factor-kappa B; PLGA, polylactic-co-glycolic acid; PARP-1, poly ADP-ribose polymerase; EGCG, epigallocatechin-3-gallate; LAD, left anterior descending; AKT, also known as protein kinase B or PKB; GSK3β, glycogen synthase kinase 3β; AMPK, adenosine 5′-monophosphate (AMP)-activated protein kinase; FGF23, fibroblast growth factor 23; HA-NB, hyaluronic acid modified o-nitrobenzyl alcohol; WPI-MA, whey protein isolate methylacrylation.

### 3.1 Astragalosides IV (AS IV)

Proinflammatory activation of vascular endothelial cells and monocytes is key to the progression of atherosclerotic events (31). AS IV, a natural saponin from the traditional Chinese medicine *Astragalus membranaceus*, ameliorates atherosclerosis by inhibiting the proinflammatory activation of endothelial cells and thus reducing monocyte infiltration (32). Mechanistically, AS IV inhibits not only inflammation-induced transforming growth factor β-activated kinase 1 mitogen-activated protein kinase (TAK1–MAPK) signaling to inhibit monocyte migration and adhesion to activated vascular endothelial cells (32), but also nucleotide-binding oligomerization domain (NOD)-like receptor thermal protein domain-associated protein 3 (NLRP3) inflammasome activation and high glucose-induced proinflammatory cytokine secretion by inhibiting Toll-like receptor 4 (TLR4)/NF-κB signaling (33). In addition, increasing evidence has shown that endoplasmic reticulum (ER) stress contributes to endothelial dysfunction, which is an early manifestation of cardiovascular disease. Although oxidative stress, inflammation and apoptosis occur simultaneously in endothelial dysfunction, ER stress is the underlying cause of these events. Under oxidative stress associated with ER stress, thioredoxin-interacting protein (TXNIP) induces NLRP3 activation, thereby promoting interleukin (IL)-1β secretion, which in turn triggers endothelial cell inflammation and apoptosis. AS IV inhibits ER stress-induced oxidative stress by regulating adenosine 5′-monophosphate (AMP)-activated protein kinase (AMPK) activity and effectively inhibits IL-1β induction by blocking TXNIP/NLRP3 inflammasome activation, thereby suppressing inflammation and protecting cell survival from ER stress (34). These results suggest that AS IV is a candidate for the treatment of atherosclerosis and can prevent vascular endothelial dysfunction, myocardial ischemia‒reperfusion injury and heart failure.

However, the bioavailability of AS IV is relatively low when it is administered orally (35). Wu Xiong et al. established a rat model of diabetic wounds and evaluated the effect of AS IV hydrogels on diabetic wounds through HE staining and Masson staining. The results showed that hydrogels exhibited good biocompatibility and promoted angiogenesis, thereby accelerating the healing of diabetic wounds (36). AS IV-loaded hydrogels could also achieve continuous and stable delivery of AS IV to the infarct area to improve its bioavailability, significantly increasing the expression of B-cell lymphoma 2 (Bcl2) protein (an antiapoptotic protein) and inhibiting the expression of apoptotic proteins such as Bcl-2-associated X-protein (Bax) and cleaved caspase 3 to inhibit apoptosis. The transcription factors involved in the pathological process of AMI include collagen type 1 and type 3, which are involved in fibrosis, and VEGF and angiopoietin (Ang)-I, which are involved in angiogenesis. In rats, the intramyocardial injection of a hydrogel loaded with AS IV significantly reduced the transcript levels of collagen 1 or 3 and increased the levels of VEGF and Ang-I mRNA, which effectively promoted angiogenesis after AMI. In addition, AS IV promotes electrical signaling in the myocardium, thus delaying ventricular remodeling and reversing impaired cardiac function (27). Therefore, this bioactive injectable conductive hydrogel may represent an effective therapeutic strategy for the treatment of AMI.

### 3.2 Triptolide (TPL)

Triptolide (TPL), composed of compounds extracted from the Chinese herbal medicine *Tripterygium wilfordii*, is famous for its anti-inflammatory and anti-apoptotic, antitumor and immunomodulatory properties (37). In clinical experiments, TPL significantly reduced cardiac inflammation and fibrosis by inhibiting NF-κB activity and expression, thereby improving left ventricular function in patients with diabetic cardiomyopathy (38). However, TPL has limited water solubility, a limited therapeutic window, and toxic effects on the urogenital system, bone marrow, digestive system, reproductive system, and blood circulation. Long-term use of TPL under complex circumstances can cause significant side effects, such as liver damage and increased serum superoxide dismutase levels (39).

Therefore, some scientists have developed and synthesized TPL hydrogels, which are expected to exhibit the continuous release of TPL and exert corresponding synergistic and detoxifying effects. Wang Fengnan et al. developed a thermosensitive hydrogel rich in TP-P1 (the precursor drug of TPL), which significantly alleviated the inflammation and oxidative stress in arthritis mice by regulating the TLR4/NF-κB pathway, significantly alleviated the symptoms of arthritis, but no obvious liver or kidney toxicity was observed (40). In a rat model of AMI, hydrogel loaded with TPL was injected into the myocardium. The results suggested that the design of the TPL hydrogel not only ensured slower and more stable TPL release but also promoted TPL retention in the myocardium. In addition, the chronic inflammatory process after AMI, which is the basic underlying pathological process, significantly affects the recovery of the infarcted myocardium, and TPL hydrogels can be used to treat AMI by inhibiting the inflammatory process (28). Connexin 43 (Cx43) is a connexin that is expressed in myocardial tissues and is required for normal cardiac conductivity. The hypoxic microenvironment in infarct tissue promotes the degradation of Cx43, which can lead to cardiac arrhythmias (41). TPL hydrogels can maintain stable Cx43 expression in infarct tissue and thus protect the myocardial structure. By the same mechanism as AS IV hydrogels, TPL hydrogels can reduce the expression of cleaved caspase 3 and decrease cardiomyocyte apoptosis. Most importantly, TPL hydrogels prevent ventricular dilation and wall thinning, which represent the anatomical basis for the recovery of cardiac function (28). These results suggest that TPL hydrogels have long-term effects supporting recovery and have potential as biomaterials for the treatment of AMI.

### 3.3 Salvianolic acid B (SalB)

*Salvia miltiorrhiza*, a kind of traditional Chinese medicine, is widely used in China for the treatment of various clinical diseases related to microcirculatory disorders, such as cardiovascular diseases, cerebrovascular diseases, renal insufficiency, liver fibrosis and diabetic vascular complications (42). SalB is the main water-soluble component extracted from *Salvia miltiorrhiza* and has a strong antioxidant effect. Recent evidence has shown that SalB has anti-inflammatory, antiapoptotic and antifibrotic effects and can promote stem cell proliferation and differentiation, with positive effects on cardiovascular and cerebrovascular diseases, aging and liver fibrosis (43). Low-density lipoprotein (LDL) can be oxidized by reactive oxygen species (ROS) to oxidized LDL (ox-LDL), which stimulates endothelial cells to secrete various inflammatory factors and promotes the adhesion and migration of monocytes into the intima of the arteries in atherosclerosis. Sal B can inhibit the production of ox-LDL, inhibit the aggregation of macrophages and reduce the uptake of ox-LDL by macrophages through antioxidant effects (44, 45). Poly ADP-ribose polymerase (PARP-1) is an abundant nuclear protein that promotes DNA base excision repair. Overactivation of PARP-1 leads to extensive cell death under pathophysiologic conditions. In addition, increased PARP-1 activation is associated with the pathogenesis of acute and chronic myocardial dysfunction. PARP-1 inhibitors have been shown to reduce myocardial remodeling more effectively than enalapril (an angiotensin-converting enzyme inhibitor, which is used clinically for lowering blood pressure and reversing ventricular remodeling, etc.) does. As higher PARP-1 expression levels are associated with disease progression, the reduction of PARP-1 expression by Sal B treatment in AMI model rats prevented further impairment of cardiac function (46).

However, owing to its inherent instability, nonspecific distribution in the body and short half-life, SalB has low efficacy and is underutilized as a drug (47). A study developed an injectable reactive oxygen species (ROS) responsive hydrogel patch, which encapsulated ROS-sensitive liposomes of SalB and enabled local and on-demand drug release in response to the oxidative microenvironment. *In vitro* studies demonstrated that this hydrogel exhibited excellent mechanical strength, selective myocardial adhesion, and sustained antioxidant capacity. *In vitro* cell experiments and mouse myocardial infarction models confirmed that the hydrogel patch could regulate macrophage polarization, stabilize mitochondrial function, activate mitochondrial autophagy, and enhance the antioxidant defense system, ultimately significantly improving cardiac function, while promoting angiogenesis, inhibiting fibrosis, and preventing cell apoptosis, ultimately significantly improving cardiac function (29). These functions of the SalB hydrogel in protecting cardiomyocytes and alleviating inflammatory responses give it great potential for clinical application. This synergistic therapeutic strategy, which involves the *in situ* administration of injectable hydrogels, may represent a new option for the treatment of AMI.

### 3.4 Rhein

Rhein is a compound with anti-inflammatory activity extracted from the herb Huang Da. Rhein promotes the synthesis of a complex involving peroxisome proliferator-activated receptor γ (PPARγ), NF-κB and histone deacetylase 3 (HDAC3), thereby blocking the acetylation of NF-κB, inhibiting the release of downstream inflammatory cytokines, and reducing inflammation (48). In addition, the AKT (also known as protein kinase B or PKB)/glycogen synthase kinase 3 β (GSK3β) signaling pathway plays a critical role in protecting the heart. Rhein can reduce apoptosis and upregulate the phosphorylation of AKT and GSK3β to strengthen the oxidative defense system, thus contributing to myocardial protection (49). Furthermore, rhein significantly inhibits fibroblast growth factor (FGF) 23 expression through the activation of AMPK, suppresses Ang II-induced cardiac hypertrophy and fibrosis, and improves cardiac systolic dysfunction through the AMPK/FGF23 axis. Rhein can also significantly reduce Ang II-induced ROS and superoxide production (50), but its clinical application is limited by its low water solubility, poor absorption, short half-life, difficulty in determining the optimal therapeutic time window, and unsatisfactory stability, as well as the potential for liver and kidney toxicity after oral administration (51).

Designed to overcome these limitations, rhein hydrogels scavenge ROS and inhibit the TLR4/NF-κB signaling cascade to reduce cardiomyocyte apoptosis under conditions of oxidative stress and inflammation. *In vivo* experiments revealed that rhein hydrogels could regulate the microenvironment in myocardial ischemia‒reperfusion by reducing ROS levels and shifting the inflammatory phase toward the repair phase, effectively improving cardiomyocyte survival. Ultimately, rhein hydrogels significantly restored cardiac function and reduced fibrosis (30). These results suggest that rhein hydrogels are an effective injectable drug delivery system for the treatment of myocardial injury.

## 4 Growth factor-loaded hydrogels

The above content states that the drugs have successfully delivered to the injured myocardial tissue, promoting improvement in cardiac function. Now, we focus on growth factor-loaded hydrogels and their potential for treating damaged hearts following AMI (Table 2).

**Table 2:** Growth factors-loaded, cells -loaded or miRs-loaded into hydrogels and their effects and mechanisms.

Hydrogels	Animal model	The time of delivery	The duration of treatment	Effects/mechanisms	References
bFGF hydrogels	Sprague-Dawley rats	30 min after LAD ligation	28–30days	1. Stimulate cardiac angiogenesis. 2. Inhibit myocardial tissue fibrosis. 3. Improve cardiac function.	(52, 53–55)
IGF-1/silk fibroin microspheres/ hydrogels	rats	–	28days	1. Reduce fibrosis length, and infarct size. 2. Attenuate cardiomyocyte apoptosis and improve cardiac function.	(56)
NRG hydrogels	Dorset sheep	10 min after LAD ligation	8 weeks	1. Induce cardiomyocyte regeneration and repair. 2. Improve ventricular function. 3. Protect cells from apoptosis. 4. Promote angiogenesis.	(57, 58)
ADSC/ASS hydrogels, MSCs hydrogels	Mice	–	28days	1. Promote re-epithelialization and angiogenesis. 2. Reduce apoptosis, oxidative stress and fibrosis. 3. Initiate immune regulation and anti-inflammatory modulation. 4. Prevent damaging remodeling.	(14, 15, 59)
hiPSCs hydrogels	Mice	–	–	1. Form more cellular aggregates and more mature gap junctions. 2. Promote angiogenesis and inhibit apoptosis. 3. Improve cardiac function and ventricular remodeling.	(60)
TeEVs hydrogels	Mice	30 min after LAD ligation	21days	1. Promote cardiomyocyte growth and proliferation. 2. Protectheart against pathological remodeling.	(61)
miR-199a-3p/nanoparticles hydrogels	rats	–	3months	1. Stimulate the proliferation of human ESC-derived cardiomyocytes and endothelial cells. 2. promote angiogenesis under hypoxic condition. 3. Decrease the scar size and the capillary density in the boundary zone	(62)
miR-29B hydrogels	Mice, sheep	–	5 weeks	1. Increase vascular distribution of myocardial scars alleviate fibrosis. 2. Improve the adverse microenvironment of myocardial infarction 3. Prevent further apoptosis of myocardial cells	(63, 64)

bFGF, basic fibroblast growth factor; LAD, left anterior descending; IGF-1, insulin-like growth factor 1; NRG, neuregulin; MSCs, mesenchymal stem cells; ADSCs, adipose-derived stem cells; ASS, alginate/silk sericin; MSCs, mesenchymal stem cells; hiPSCs, human induced pluripotent stem cells; TeEVs, targeting miR-222-engineered extracellular vesicles.

### 4.1 bFGF

Collateral blood flow through the coronary artery may be sufficient to maintain wall motion and prevent ischemia at rest, and it may reduce or prevent ischemia during exercise. Since an increased number of collateral vessels may be due to the formation of new vessels, the promotion of coronary collateral growth represents a new and potentially important therapeutic approach for patients with ischemic heart disease (65). bFGF, a potent mitogen that regulates angiogenesis during growth and development, has been successfully used to stimulate cardiac angiogenesis in various animal models of myocardial ischemia to improve the myocardial blood supply (66). bFGF also directly affects tissue fibrosis by inhibiting the TGF-β-induced differentiation of fibroblasts into myofibroblasts. However, the biological half-life of bFGF is too short—reportedly less than 50 min—and the biological effects of the protein-free form of bFGF are very limited (67). *In vitro*, immediately injecting bFGF loaded hydrogels into the myocardium after myocardial infarction in rats can significantly reduce the infarcted area, lasting for up to 30 days. A bFGF release system with appropriate release kinetics prevented cardiac fibroblasts from differentiating into myofibroblasts, stimulated endothelial cell regeneration, promoted microvessel growth in the early phase of AMI, inhibit cardiomyocyte apoptosis, alleviate ventricular remodeling, promoted cardiac fibroblast survival and improve cardiac function. *In vivo*, the bFGF release system significantly also reduced cardiac fibrosis, promoted angiogenesis and improved cardiac function (52–55). These results suggest that bFGF hydrogels can be used as a new therapeutic approach for the treatment of AMI.

### 4.2 Insulin like growth factor (IGF-1)

IGF-1 is the most important growth factor for somatic cells before puberty and adolescence and mediates many of the functions of growth hormone (68). IGF-1 affects vascular function and atherosclerosis in several ways, including as an anti-inflammatory and anti-apoptotic factor (69) and as a stimulator of angiogenesis (70). Local IGF-1 expression in cardiomyocytes protects the heart from oxidative stress and promotes functional recovery after AMI (71). However, IGF-1 infusion into the systemic circulation limits its efficacy due to its short half-life and low uptake in the myocardium (72). To avoid these problems, the continuous administration of IGF-1 has been shown to be a valuable alternative strategy. Jianguo Feng et al. prepared silk fibroin (SF) microspheres, physically adsorbed IGF-1 onto the SF microspheres and then loaded these microspheres into alginate salt gels. The results revealed that this composite hydrogel can promote the proliferation of H9C2 cardiomyocytes and reduce the apoptosis rate under hypoxic conditions. The results of enzyme-linked immunosorbent assay indicate that SF microspheres, as microcarriers, can effectively enhance the sustained release of IGF-1 from the hydrogel, making the composite hydrogel have a superior sustained release ability compared to the system without SF microspheres. Additionally, the results of echocardiography, hematoxylin-eosin staining, and Masson trichrome staining show that injecting the composite hydrogel into the peripheral region of the myocardial infarction rat model can increase the ventricular wall thickness 28 days later, significantly reduce the length of the fibrotic area, minimize the infarction area, and improve cardiac function. Compared with the control group, the effect is significant (56). These results demonstrate that the SF/IGF-1 hydrogel can effectively attenuate cardiomyocyte apoptosis in the AMI environment and improve cardiac function after AMI, with a direct beneficial effect on cardiomyocytes.

### 4.3 Neuregulin (NRG)

NRG, a signaling growth factor released by endothelial cells in the ventricular endocardium and cardiac microvasculature, is a ligand for the ErbB4 tyrosine kinase receptor (also known as epidermal growth factor receptor 4), which activates intracellular signaling cascades leading to proliferation, differentiation, and migration and is among the most promising bioactive factors for cardiac regeneration (73). Previous reports have shown that NRG-1/ErbB signaling can effectively induce cardiomyocyte proliferation, protect cells from apoptosis, promote angiogenesis, and support cardiac regeneration and repair by activating extracellular signal-regulated protein kinase (ERK) and AKT signaling pathways (74–76). In some clinical cases, patients with chronic heart failure have been treated with NRG and achieved sustained improvements in cardiac function (57). However, the therapeutic efficacy of bioactive agents is usually limited because of their low retention rates. In a mouse model of ischemic cardiomyopathy, treatment with NRG hydrogels resulted in reduced left ventricular dilation, significant improvement in left ventricular ejection fraction (LVEF) and increased junctional zone thickness 2 weeks after treatment. The *in vitro* and *in vivo* data showing the mitotic activity of cardiomyocytes demonstrate that the NRG hydrogel induces cardiomyocyte regeneration and contributes to improved ventricular function. The reduced level of activated caspase 3 after treatment with the NRG hydrogel also indicates its antiapoptotic effect (57). To further evaluate the potential of the NRG loaded hydrogel therapy in enhancing cardiac function in sheep myocardial infarction model. Sheep were randomly assigned to receive saline, only hydrogels, only NRG, or NRG-HG myocardial injection around the infarction border. The experimental results were observed eight weeks after myocardial infarction. Compared with each control group, NRG loaded hydrogel significantly increased the LVEF (*p* = 0.006) and contractility, mainly based on the slope of the pressure-volume relationship at the end of contraction (*p* = 0.006), and significantly reduced the infarction scar area (*p* = 0.002) (58). Overall, these results suggest that treatment with NRG hydrogels leads to improved ventricular function and structure.

## 5 Cell-loaded hydrogels

Stem cells or stem cells-loaded exosomes have shown excellent therapeutic effects in treating heart failure (77, 78), but it is estimated that over 90% of the stem cells infused into the heart fail to reach the heart. This occurs for two reasons. Firstly, due to the abundant blood flow within the heart, the infused stem cells are quickly cleared and cannot remain for a long time to exert therapeutic effects. Secondly, stem cell therapy should be initiated immediately upon the occurrence of acute injury. However, during acute injury, there is a strong immune and inflammatory response in the infarcted area of the heart (79). Therefore, the stem cells injected into this area will be in a harsh environment, resulting in a reduced cell survival rate.Therefore, we summarize the efficacy and mechanism of combining stem cells with hydrogels for the treatment of heart failure to increase intracardiac retention and cell survival rate (Table 2).

### 5.1 Mesenchymal stem cells (MSCs)

MSCs are multipotent stem cells that can be isolated from bone marrow (BM), adipose tissue, umbilical cord blood, bone, myocardium, the pancreas and many other tissues. However, the two most common sources for clinical applications are bone marrow (bone marrow-derived MSCs, BM-MSCs) and adipose tissue (adipose-derived stem cells, ADSCs). Bone marrow is easily renewed, and fat is usually abundant or unused, making BM-MSCs and ADSCs ideal candidates for regenerative medicine (80). Key mechanisms of action include promoting re-epithelialization and angiogenesis (81, 82), reducing apoptosis and oxidative stress (e.g., in the heart or kidneys) (83, 84), reducing fibrosis in tissues (e.g., in the heart) (83), initiating immune regulation and mitigating inflammation (85).

Clinical studies have shown that MSCs are usually injected systemically or locally into target tissue. The limited reparative effect of MSCs on damaged myocardium is related mainly to the low homing rate and survival rate of the cells. Fewer than 1% of cells survive 24–48 h after injection, which poses a challenge for achieving sustained therapeutic effects (86). The use of hydrogel-encapsulated MSCs is a promising approach for the treatment of AMI. Compared with direct MSC transplantation, an injectable ADSC-loaded hydrogel (ADSC-hydrogel) increased cell survival and the expression of angiogenic and anti-inflammatory factors *in vitro* and protected cells from oxidative damage in the infarct area as well as other survival-reducing stimuli. The ADSC-hydrogel also promoted cell proliferation and division, enhanced angiogenesis and the transport of oxygen and nutrients, improved cell communication, preserved cardiomyocyte function and regulated immunity, thereby effectively and successfully treating cardiac injury after AMI (14, 15).

Human umbilical cord-derived MSCs (hUC-MSCs) are also promising therapeutic candidates for myocardial engineering because they have unique advantages over other sources, such as ease of isolation and collection, lack of ethical concerns, low immunogenicity, and the possibility of producing standard biopharmaceuticals (87). He Xiaojun et al. selected 50 patients with LVEF of 45% or lower to undergo elective coronary artery bypass grafting (CABG), and randomly divided them into the hUC-MSCs loaded hydrogel treatment group (hydrogel group), the simple cell treatment group, or the control group. During the CABG procedure, patients in the hydrogel group received intramyocardial injection of collagen gel containing hUC-MSCs, while the cell group received simple hUC-MSCs treatment. Patients in the control group only received the CABG surgery. At the baseline stage, there were no differences in the characteristics of the patients in each group. For the primary endpoint indicators, no serious adverse events, myocardial injury indicators, or significant differences in renal or liver function were observed in all treatment groups after treatment; 1 patient in the hydrogel group and 1 patient in the cell group were hospitalized due to heart failure, while no serious adverse events occurred in the control group. After 12 months of treatment, a 3.1% reduction in infarct size after the myocardial injection of a collagen hydrogel loaded with hUC-MSCs compared with a 5.2% increase after the myocardial injection of hUC-MSCs alone (59). Hydrogel encapsulation provides a new avenue for the application of MSCs in AMI treatment, improves cell therapy technology and is an effective candidate for AMI treatment.

### 5.2 Human induced pluripotent stem cells (hiPSCs)

Like human embryonic stem cells (ESCs), hiPSCs are a promising source of repair cells in the heart, as they have the capacity for unlimited self-renewal and differentiation into cardiac muscle cells (88). hiPSCs and their derived cardiomyocytes (hiPSC-CMs) have shown considerable potential for cardiac regeneration in large rodent models (89). However, functional and phenotypic immaturity severely limits the recruitment of damaged cardiomyocytes and the restoration of electrical conduction in the injured myocardium by hiPSC-CMs (90). The relatively low expression of gap junction proteins, particularly Cx43, in hiPSC-CMs results in poor electrical integration between the host myocardium and the cell graft, which may pose an arrhythmogenic risk to the host heart (91). Therefore, the maturation of hiPSC-CMs needs to be enhanced to form functional syncytia with the host myocardium before clinical translation. *In vivo*, hiPSC-CMs delivered in a hydrogel formed more cellular aggregates and more mature gap junctions in the infarct-like myocardium of mice after transplantation. In addition, hiPSC-CMs encapsulated in the hydrogel showed more durable angiogenic and antiapoptotic effects in the peri-infarct area. These biological properties of hiPSC-CMs improve overall cardiac function after AMI and enhance ventricular remodeling (60), representing a new approach for cardiac repair after AMI based on the transplantation of hiPSC-CMs.

## 6 Exo-loaded hydrogels

Exos are nanoscale, membrane-bound extracellular vesicles that are secreted by cells and mediate the intercellular transport of cytoplasmic goods (92). Stem cell-derived exos are rich in a variety of functional proteins and cytokines and have the same cardioprotective effects as their parent cells do (93). The implantation of stem cells into the myocardium presents numerous challenges, including immune rejection, limited cell survival, and the risk of ventricular arrhythmias. Compared with the implantation of stem cells into the myocardium, research on the therapeutic effects of these cell-derived extracellular vesicles in ischemic hearts has shown superior efficacy. These vesicles do not require cell implantation, reducing the potential tumorigenicity and immunogenicity of stem cell transplantation, and can be easily manufactured using biopharmaceutical standards (94). However, the half-life of exos in organs is relatively short (70–80 min), and systemic administration readily results in rapid clearance of exos by phagocytes, leading to preferential accumulation in organs of the mononuclear phagocyte system, such as the lungs, spleen and liver, resulting in uncontrollable side effects and biosafety hazards (95). Additionally, multiple doses of intramyocardial injections are cumbersome and time-consuming for surgery (96). These obstacles significantly limit the clinical application of exosome-based drugs. Numerous studies have shown that the administration of exo-loaded hydrogels can effectively prolong the dwell time and improve the local application of exos (97–99). Therefore, we investigated the possibility of combining hydrogels and exos to treat myocardial injury (Table 3).

**Table 3:** Different exos-loaded into hydrogels and their mechanisms and effects.

Hydrogels	Animal model	The time of delivery	The duration of treatment	Effects	References
ADSC-exos hydrogels	Mice	15 min after LAD ligation	21days	1. Promote angiogenesis. 2. Reduce cardiac remodeling.	(100)
hUC-MSC-exos hydrogels	–	–	–	1. Promote cell survival. 2. Reduce inflammation and fibrosis. 3. Inhibit apoptosis. 4. Further promote angiogenesis.	(101)
DC-exos hydrogels	Wild-type mice	–	14days	Improve cardiac function.	(102)
MSC-exos/ hyaluronic acid hydrogels	Rats	Immediately after LAD ligation	28days	1. Regulate immune responses. 2. Enhance proliferation of cardiomyocytes and reduce apoptosis. 3. Reduce interstitial fibrosis. 4. Promote angiogenesis.	(98, 103)

ADSC-exos, adipose tissue-derived MSC-derived exos; LAD, left anterior descending; hUC-MSC-exos, human umbilical cord-derived MSC-derived exos; DC-exos, dendritic cell-derived exos; MSC-exos, mesenchymal stem cell-derived exos.

### 6.1 ADSC-derived exos

Mesenchymal stem cells derived from adipose tissue have been shown to increase survival rates, reduce inflammation and accelerate wound healing (104). However, stem cell therapy for ischemic heart disease has several risks. The main obstacles are the low survival rate and high embolization risk of cells transplanted into the ischemic area, as well as endogenous factors such as the recipient's inflammatory response. Fortunately, ADSC-derived exos can replicate the anti-inflammatory, antiapoptotic, proangiogenic and antifibrotic effects of their parental cells and thus improve cardiac function (105). In addition, as alternatives to ADSCs, exos can prevent the low survival rate, immune rejection and tumorigenicity caused by stem cell transplantation (106). Researchers have successfully developed an injectable therapeutic nanocomposite hydrogel that promotes angiogenesis *in vitro* and *in vivo*, reduces cardiac remodeling, and protects the heart in a rat model of AMI by injecting ADSC-derived exos into the peri-infarct region (100). Overall, these results suggest that stem cell-derived exo-loaded hydrogels are a viable option for promoting myocardial regeneration and myocardial therapy after AMI.

### 6.2 Human umbilical cord mesenchymal stem cell-derived exos (hUC-MSC-exos)

The presumed mechanism underlying stem cell-based therapies has fundamentally shifted from cell differentiation to paracrine effects that involve endogenous repair mechanisms (107). A conductive and injectable hydrogel system was constructed for the sustained release of hUC-MSC-exos to restore cardiac function after AMI. The results of histological analysis, immunofluorescence staining and reverse transcription‒polymerase chain reaction showed that the hydrogel alone promoted the repair of cardiac biological structure and function, indicating that the prepared hydrogel had a biological effect. The results of animal experiments have shown that the synergistic effects of a conductive, bioavailable hydrogel with exosome-anchoring properties and hUC-MSC-exos can optimally repair myocardial injury by significantly upregulating heart-related proteins and angiogenic factors, which in turn improves cell‒cell interactions and promotes cell proliferation and angiogenesis in the injured heart (101). Taken together, these results provide an effective platform for the treatment of ischemic cardiomyopathy, and such a system may be a promising optimization strategy with translational potential for clinical application.

### 6.3 Dendritic cell (DC)-derived exos (DC-exos)

DC-exos are known to be involved in antigen presentation (108) and immune activation and suppression (109). Recent studies have shown that in the context of the myocardial cell microenvironment after AMI, DCs secrete many exos that rapidly concentrate in the spleens of mice and act directly on CD4+ T cells. One theory suggests that exos secreted by DCs after AMI act as direct activators of CD4+ T cells via an endocrine mechanism and improve cardiac function (110). However, the intravenous administration of exos is associated with a short half-life because of clearance by monocytes and accumulation in the liver and spleen, with low retention rates significantly limiting therapeutic efficacy (111). However, simply administering more DC-exos does not prolong the retention time (112). Therefore, the development of a new method to improve the retention time of DC-exos is clearly needed. Some scientists have combined DC-exos with alginate hydrogels (DC-exo gels) to investigate the controlled release ability of DC-exos. They reported that DC-exo gels can sustainably release and prolong the residence time of DC-exos but do not affect the migration of DC-exos *in vivo*. DC-exo gels were then applied to AMI model mice, where the gel significantly enhanced the therapeutic effects of DC-exos on improving cardiac function after AMI. Flow cytometry and immunofluorescence staining revealed that DC-exos significantly increased the infiltration of regulatory T cells and M2 macrophages after AMI *in vitro* and *in vivo*, activated regulatory T cells, and converted macrophages into reparative M2 macrophages (102).

### 6.4 MSC-derived exos (MSC-exos)

Compared with MSCs, MSC-exos share similar protein factors, lipid messengers and regulatory microRNAs (miRNAs) and induce their therapeutic effects mainly by triggering regenerative and antifibrotic signaling pathways mediated by various transport miRNAs (113). MSC-exos play important roles in regulating immune responses (114) and reducing cardiomyocyte apoptosis through miR-199a-3p (115). This mechanism involves enhanced proliferation and reduced apoptosis of cardiomyocytes (116). However, intravenously administered exos are rapidly cleared from the blood and accumulate in organs such as the brain, lungs and liver but not the heart. An injectable hyaluronic acid hydrogel loaded with MSC-exos has been developed and introduced into the pericardial space of rodent or pig hearts using a minimally invasive thoracoscopic procedure. The therapy reduced the size of the left ventricle compartment and the extent of interstitial fibrosis; it also preserved the wall thickness, protected cardiomyocytes from hypertrophy, promoted cardiomyocyte proliferation and reduced cardiomyocyte apoptosis (98).

Certain researchers have further investigated injectable hydrogels capable of gradually releasing oxygen, aiming to address the limitations associated with current oxygen therapies for AMI. These hydrogels accumulate in the infarct regions of the heart and facilitate oxygen release during the early phases of AMI. O_2_ (+) hydrogels, which are coated with exos derived from MSCs, release substantial quantities of oxygen for more than five days under hypoxic conditions. Following seven days of *in vitro* culture, the injured cardiomyocytes exhibited paracrine factor production comparable to that observed in cultures of rat cardiac fibroblasts, rat neonatal cardiomyocytes, and human umbilical vein endothelial cells, indicating their ability to mimic the native structure and function of capillaries. In an AMI rat model, cardiomyocytes located in the peri-infarct region treated with the Exo-O_2_ (+) hydrogel demonstrated significant mitogenic activity four weeks post-treatment. Furthermore, compared with those treated with the O_2_ (−) hydrogel, hearts subjected to the Exo-O_2_ (+) hydrogel exhibited a notable increase in the myocardial capillary density (103).

Collectively, these findings illustrate that the exo-loaded hydrogel offers protection to the heart against the deterioration of pumping function and morphological changes through its beneficial effects on hypertrophy, proliferation, and apoptosis, further validating the feasibility and safety of exo-loaded hydrogel injection in a porcine model.

## 7 miRNA-loaded hydrogels

miRNAs are short, 21- to 22-nucleotide-long, double-stranded RNAs that are physiologically generated by the cellular RNA-induced silencing complex by processing longer hairpin RNAs, which are, in turn, the processing products of upstream enzymes of the RNA interference machinery. The intracellular overexpression of miRNAs in cardiomyocytes can be effectively achieved by transferring the respective coding genes using adeno-associated virus (AAV) vectors, which efficiently transduce cardiomyocytes after either intracardiac or systemic delivery. AAV vectors persist episomally and express their transgenes for prolonged periods, virtually coinciding with the life of the organism (117). In addition, a portion of the injected vectors also transduces other organs, in particular, the liver and skeletal muscle, in the case of the AAV9 serotype (118). Finally, because miRNA production after the virus-mediated delivery of coding genes depends on the endogenous RNA interference machinery for processing, either miRNA strand can be used according to the expressing cell type. These characteristics raise both safety and efficacy concerns. Therefore, we considered the use of hydrogels to deliver miRNAs to overcome these shortcomings, such as miR-199a-3p miR-29B and miR-222 (Table 2).

### 7.1 miR-199a-3p

miR-199a-3p has demonstrated considerable therapeutic potential in facilitating cardiovascular regeneration through enhancing the proliferation of cardiomyocytes derived from mice and rats through its molecular targets, Homer scaffold protein 1 and chloride intracellular channel 5, as well as promoting the proliferation of rat endothelial cells via caveolin 2 (119–121). Additionally, miR-199a-3p has been shown to mitigate lung and kidney fibrosis by activating p-AKT survival signaling through carvedilol (122) and to provide cardioprotection in ischemic cardiomyopathy by inhibiting TGF-β signaling pathways associated with FGF7 and HGF (123). To improve the feasibility of miRNA gene therapy for future clinical applications, various *in vivo* delivery methods, including the utilization of AAVs, have been investigated. Despite the high transfection efficiency of AAVs observed in numerous *in vitro* and *in vivo* studies, their clinical application is hindered by concerns regarding potential immune responses (124, 125), long-term transgene expression, and the absence of spatiotemporal control (126). Researchers have developed an *in vivo* delivery system employing polymer nanoparticles encapsulating miRNAs (miNPs) for localized delivery within shear-thinning injectable hydrogels. These miNPs have been shown to stimulate the proliferation of human ESC-derived cardiomyocytes and endothelial cells and to promote angiogenesis under hypoxic conditions, resulting in significantly lower cytotoxicity than that of Lipofectamine. Furthermore, a single administration of the hydrogel/miNP complex in AMI rat models resulted in marked improvements in cardiac function, with the ejection fraction increasing from 45% to 64%, the scar size decreasing from 20% to 10%, and the capillary density in the boundary zone doubling relative to those in control groups (62). Consequently, this injectable hydrogel/miNP system represents an effective and safe method for delivering miRNA to facilitate the repair of damaged myocardium.

### 7.2 miR-29B

At least 16 genes related to the ECM are targeted by members of the miR-29 family. These genes encode several key proteins involved in the physiological or pathological formation of the ECM, including numerous collagen subtypes, laminin γ, fibrin 1, elastin, MMP-2, and integrin β1 (127). miR-29B is preferentially expressed in fibroblasts and is related to the regulation of fibrosis in many tissues, including hepatic (128) and cardiac tissues (129). However, in several pathological tissue sections, the expression of miR-29B was found to be downregulated around the infarct area. This change plays an important role in ECM remodeling and is significantly related to collagen production (130, 131). The downregulation of miR-29 promotes the development of cardiac fibrosis by alleviating its inhibitory effect on the expression of ECM-related genes (129). In fact, miR-29 may also be involved in the apoptotic process. miR-29 can trigger fibroblast apoptosis by targeting the cell division cycle (42). At the end stage of heart failure, when ECM deposition is very extensive in the myocardium, the role of miR-29 in apoptosis may be more important than that in fibrosis (131).

An injectable hyaluronic acid-based hydrogel was used to locally deliver miR-29B to the boundary area of the infarction. After the surgery, the myocardial function of the animals was monitored for up to 5 weeks. In this proof-of-principle study, the myocardial function in the experimental group remained normal at 2 weeks and 5 weeks after myocardial infarction. Additionally, the myocardial function of the animals treated with the nontargeting miRNA control using the hyaluronic acid-based hydrogel significantly deteriorated, whereas the animals treated with miR-29B did not show significant deterioration. Histological analysis revealed that in the infarct boundary area, compared with that in the nontargeted miRNA treatment group, the number of green immature fibers in the miR-29B treatment group was significantly lower, and the number of red‒orange, more mature collagen fibers was significantly greater. These mature collagen fibers were stronger and arranged around the myocardial wall, providing compensatory support for the infarcted myocardial wall. The vascular distribution of myocardial scars also increased, and Raman microspectroscopy revealed subtle but significant changes in ECM tissue and maturity (63). Recently, an injectable alginate composite hydrogel has been developed. Specifically, mesoporous silicon nanoparticles (MSNs) are used as carriers to encapsulate miR-29B mimics, and then coated with a TA/Zn complex, thereby forming nanoparticles named TMR NPs. When this composite hydrogel is injected into the infarcted area, first, the TA/Zn complex on the outer layer of TMR NPs acts as an antioxidant to inhibit cell apoptosis. Subsequently, as the TA/Zn complex dissociates, the encapsulated miR-29B mimics are released to alleviate fibrosis. Moreover, the released Zn2 + promoted angiogenesis in the infarcted area, thereby improving the adverse microenvironment of myocardial infarction and preventing further apoptosis of myocardial cells (64). This preclinical proof-of-principle study demonstrated not only that the miR sequence is the main reason for the improvement in cardiac function but also that the injectable hyaluronic acid hydrogel system may be able to deliver miR-29B to maintain cardiac function after MI and has great potential for the local treatment of ischemic tissues using injectable biomaterials such as exogenous miRNAs to regulate tissue remodeling.

### 7.3 miR-222

Regarding the role of miR-222 in the structural and functional responses of the heart to continuous pathological stress, the evidence is contradictory. On one hand, F. Janse et al. identified miRNAs in the peripheral blood of patients with significant coronary artery stenosis (>80% diameter stenosis), and in these patients, miRNA-21, miRNA-126-3p, and miRNA-222 in the circulation increased under the stress response of the heart (132). Karere G.M. et al. identified novel miRs that might be related to atherosclerosis, with increased expression of miR-144-3p, miR-146a-5p, miR-21, and miR-221/222-3p in fibrous plaques (133). According to the current scientific data, miR-222 is involved in the control processes of endothelial inflammation, angiogenesis, and endothelial cell apoptosis, such as through the overexpression of miR-19b-3p, miR-221-3p, and miR-222-3p induced by inflammatory cytokines TNF-α and IFNγ, leading to the accumulation of intracellular ROS and subsequently triggering cell apoptosis. In obstructive coronary artery disease (CAD), there is a moderate correlation between miR-222 and TNF-α expression, and it is highly correlated with MMP-1 and MMP-14 in various zinc-dependent endopeptidases (MMPs are synthesized in an inactive form and regulated by various pro-inflammatory factors) (134). On the other hand, to evaluate the potential protective effect of overexpressing miR-222 on ischemia-reperfusion injury (IRI), researchers let mice overexpressing miR-222 undergo IRI caused by 30 min of coronary artery ligation and reperfusion. One day after the ischemic injury occurred, miR-222 overexpression mice did not differ from the control group in terms of the initial infarct area and cardiac function. However, the control group mice showed typical ventricular gradual dilation and decreased cardiac function, while miR-222 overexpression mice did not have such changes but maintained the size of the ventricle and cardiac function. Six weeks after the ischemic injury, miR-222 overexpression mice had better function and a 70% reduction in cardiac fibrosis. The proliferation marker pHH3 in the cardiac myocytes of miR-222 overexpression mice increased by two times one week after ischemic injury compared to the control group. In contrast, the apoptosis of cardiac myocytes in the hearts of overexpressing miR-222 mice decreased by three times at the same time point, and there was no significant difference in the expression of hypertrophy markers after ischemic injury (135).

The extracellular vesicles (TeEVs) engineered for miR-222 were combined with injectable pericardial hydrogel patches. The GEL-TeEV heart patch was injected into the pericardial cavity using a minimally invasive method, and the TeEVs in the hydrogel released rapidly within the first 24 h, enhancing the rescue effect of acute IRI. Slowly controlled TeEVs released within 3 weeks alleviated the cardiac dysfunction after IRI remodeling. TeEVs reduced myocardial apoptosis, cardiac fibrosis, and immune inflammatory responses. More TeEVs alleviated myocardial fibrosis three weeks after IRI remodeling, thereby reducing cardiac mechanics. In conclusion, targeted and engineered TeEVs were released controllably from the mechanical hydrogel patch and precisely delivered to the cardiac myocytes, through regulating mechanical signal activation, assembly of adhesion-related proteins, opening of cytoskeletal force channels, and nuclear mechanical transduction, alleviating ischemic myocardial disease (61). Therefore, the therapeutic effect of the miR-222 loaded hydrogel in treating myocardial injury still requires further experimental verification.

## 8 Dual substances-loaded hydrogels

The above content indicates that some researchers have explored the use of hydrogels to deliver a kind of substance for treating myocardial injury. To determine whether combining two substances carried by the hydrogel would have a synergistic protective effect on the myocardium, additional research was conducted by some scholars (Table 4).

**Table 4:** Two therapeutic agents-loaded into hydrogels and their effects and mechanisms.

Hydrogels	Animal model	The time of delivery	The duration of treatment	Mechanisms/effects	References
Curcumin/rhCol III hydrogels	rats	–	28days	1. Reduce inflammation. 2. Alleviate apoptosis. 3. Promote angiogenesis and remodeling of myocardial tissue.	(136)
rTFPI2/VEGF/MSN hydrogels	Mice	15 min after LAD ligation	28days	1. Scavenge reactive oxygen species. 2. Reduce apoptosis. 1. Modulate macrophage polarization from pro-inflammatory M1 to reparative M2 phenotypes. 2. Enhance angiogenesis. 3. Inhibit cardiac fibroblast activation. 4. Significantly reduce infarct size.	(11)
Crocin/BMSCs alginate hydrogels	Wistar rats	After LAD ligation	28days	1. Significantly reduce scar thickness. 2. Promote angiogenesis.	(137)
Colchicine/ECM/ NLCs hydrogels	Mice	–	21days	1. Increase vascular density. 2. Reduce fibrosis. 3. Enhance oxidative phosphorylation and fatty acid degradation activity.	(138)
ISL1-MSC-exos/ Angiopoietin I hydrogels	–	–	–	1. Alleviate apoptosis. 2. Promote endothelial cell survival and angiogenesis.	(139)
HGFdf/ HA/PEG-PLA hydrogels	Wistar rats, Dorset sheep	Immediately after LAD ligation	28days	1. Stimulate angiogenesis in the boundary area. 2. Preservation of the geometry and function of the left ventricle.	(140)

rhCol III, recombinant human type III collagen; LAD, left anterior descending; BMSCs, bone-derived mesenchymal stem cells; rTFPI2, recombinant tissue factor pathway inhibitor 2; VEGF, vascular endothelial growth factor; MSN, mesoporous silica nanoparticles; ECM, extracellular matrix; NLCs, nanostructured lipid carriers; ISL1-MSC-exos, islet-1 (ISL1)-MSC-derived exosomes; HGFdf, dimeric fragment of hepatocyte growth factor; ESA, engineered analog of stromal cell-derived factor 1α;HA, hyaluronic acid; PEG-PLA, poly(ethylene glycol)-block-poly(lactic acid); LAD, left anterior descending.

### 8.1 Curcumin (Cur) and recombinant human collagen type III (rhCol III)

Numerous studies in models of myocardial injury *in vitro* and *in vivo* have shown that Cur can reduce the formation of ROS, the adhesion of monocytes to endothelial cells, and the phosphorylation of p38 mitogen-activated protein kinase, signal transducer and activator of transcription 3 and downstream signaling pathways, thereby preserving myocardial function after cardiac ischemia (141, 142). Recent studies in models of pressure overload-induced cardiac hypertrophy and heart failure have shown that Cur can reduce cardiac remodeling by altering the Ang II-transforming growth factor (TGF)-β1 axis (143). However, the therapeutic potential of Cur is often limited by its low water solubility and limited bioavailability (144).

Thus, researchers prepared an injectable carrier loaded with rhCol III and Cur-loaded nanoparticles. rhCol III is composed of the highly active fragment of the human type III collagen protein (COL3A1) (145). Its immunogenicity is lower than that of collagen derived from animals. Previous studies have shown that rhCol III has the ability to promote cell activity and angiogenesis (146). First, in the Cur/rhCol III hydrogel group, the expression levels of α-actin, a typical cardiac marker related to the maturation and contraction of myocardial tissue, and Cx43 increased, and the high expression of Cx43 and α-actin indicated the formation and remodeling of gap junctions between cardiomyocytes. Second, M1 macrophages produce high levels of inflammatory cytokines (e.g., tumor necrosis factor-α (TNF-α) and IL-6), and M2 macrophages express high levels of the anti-inflammatory cytokine IL-10. The Cur/rhCol III hydrogel significantly reduced the expression of inflammatory TNF-α and IL-6 and increased the expression of anti-inflammatory IL-10. Quantitative data on infarct size also revealed the least fibrosis in the Cur/rhCol III hydrogel group (136). These results suggest that Cur/rhCol III hydrogels could be beneficial for restoring cardiac function and accelerating the repair of damaged myocardial tissue after AMI.

### 8.2 Crocin (CRO) and BM-MSCs

CRO is one of the most abundant bioactive compounds in saffron, and it has various pharmacological effects, including antioxidant, free radical scavenging, anti-inflammatory, anti-atherosclerosis, and cardioprotective effects (147). In the study by Wang et al., CRO was proven to protect myocardial cells from damage caused by myocardial infarction by promoting angiogenesis (148). In clinical trial studies, saffron and CRO were found to inhibit the appetite of patients with CAD, reduce dietary intake, alleviate central obesity, and regulate lipid levels (149). Additionally, it was discovered that saffron and CRO could upregulate the expression of Sirtuin 1 and AMPK genes, while downregulating the expression of lectin-like oxidized low-density lipoprotein receptor 1 and NF-κB. Moreover, they also had beneficial effects on the levels of ox-LDL and monocyte chemoattractant protein 1 in patients with coronary artery disease and human measurement indicators (150). However, studies have shown that CRO, when orally administered, is hydrolyzed into crocetin before or during intestinal absorption, and then metabolized into mono- and di-glucuronide conjugates, resulting in low CRO bioavailability (151). Despite its beneficial properties, the insufficient stability and high sensitivity of CRO treatment pose significant challenges for its practical application. Therefore, an injectable CRO hydrogel encapsulating BM-MSCs was developed. The heart function was evaluated by echocardiography 28 days after injection. The results showed that the CRO hydrogel could serve as an injection carrier for BM-MSCs, significantly improving the therapeutic effect of myocardial infarction. Firstly, the delivery in the form of hydrogel significantly increased the retention rate of BM-MSCs in the myocardial infarction area, and the CRO hydrogel, with its strong antioxidant properties, could effectively eliminate excess ROS in the myocardial infarction area, thereby creating the optimal growth microenvironment for transplanted BM-MSCs and host myocardial cells. Additionally, the CRO hydrogel promoted blood flow in the infarcted myocardium by promoting neovascularization. Moreover, the hydrogel provided mechanical and structural support to the damaged cardiac tissue, thereby increasing ventricular wall thickness and reducing wall stress. Finally, the gel-like characteristics of the hydrogel play a crucial role in inhibiting adverse adaptive remodeling. Alginate-based hydrogels also provide long-term mechanical support for cardiac repair due to their relatively slow degradation rate (137). The combination of CRO alginate hydrogels with BM-MSCs may become a promising therapeutic option for cardiac repair and provide valuable insights into cardiac regeneration.

### 8.3 VEGF and tissue factor pathway inhibitor 2 (TFPI2)

After coronary artery obstruction leads to AMI, reducing the damaged area during interventions may be part of potential treatments for heart failure (152). In cardiac regeneration, the formation of new blood vessels around the infarct area reduces cardiomyocyte death and thus reduces tissue damage (153). VEGF is among the most potent mediators of angiogenesis (154). One of the largest clinical trials of the use of VEGF is the Vascular endothelial growth factor in Ischemia for Vascular Angiogenesis trial. This clinical trial revealed the safety of VEGF therapy and an improvement in the quality of life among patients who received VEGF. However, myocardial perfusion did not significantly improve (155). It has been suggested that the sustained release of growth factors at the site of action for up to 4 weeks is required for maximum benefit from VEGF therapy (156). However, VEGF has a short circulating half-life and is easily degraded. This conflicts with the persistent high concentration of local VEGF required for the development of mature blood vessels. Overdose can lead to unnecessary complications, such as uncontrolled tumor formation and vascular leakage, leading to edema and hypotension (157). Tissue Factor Pathway Inhibitor 2 (TFPI2) is a broad-spectrum Kunitz-type serine protease inhibitor that has recently been proven to be a key regulatory factor in inflammatory responses, extracellular matrix integrity, and fibrotic processes (158). Previous studies have shown that TFPI2 can reduce collagen deposition and adverse fibrotic remodeling by inhibiting the expression of MMP2/MMP9 in infarcted myocardium, thereby inhibiting the degradation of extracellular matrix, inflammatory infiltration, and the migration and activation of myofibroblasts (159). Importantly, TFPI2 can also regulate the polarization of macrophages, promote the differentiation of M2-type macrophages, and inhibit excessive inflammatory responses (160). Therefore, a smart responsive hydrogel carrying VEGF and TFPI2 has been developed, which can precisely and controllably release different therapeutic agents during the inflammatory, proliferative, and remodeling stages of myocardial infarction. Firstly, the methylacryloyl carboxymethyl chitosan in the hydrogel structure has significant pH sensitivity and can respond to pH changes during the inflammatory stage after infarction, thereby exerting anti-apoptotic and anti-inflammatory effects. Secondly, the TFPI2 carried is released with the expansion/degradation of the hydrogel during the inflammatory stage and prolongs the release time, significantly improving the drug utilization efficiency. Vascular Endothelial Growth Factor (VEGF) is encapsulated in microsphere nanosystems, which have stable and continuous release characteristics that prevent the depletion of VEGF during the waiting period, allowing it to be released during the proliferative stage of myocardial infarction. *In vitro* observations show that the hydrogel clears reactive oxygen species, reduces apoptosis of H9C2 cardiomyocytes, regulates macrophage polarization, transforming from pro-inflammatory M1 to reparative M2 phenotype, inhibits secretion of IL-6 and TNF-α. It also enhances the proliferation, migration, and angiogenesis of cardiac microvascular endothelial cells (CMECs), promotes microvascular reconstruction at the infarct edge zone, and inhibits activation of cardiac fibroblasts. In mouse myocardial infarction models, the hydrogel significantly reduces infarct size, improves cardiac function, and alleviates fibrosis. Immunohistochemistry confirms enhanced vascular density, reduced expression of MMP2/MMP9, and promotion of macrophage polarization to M2 phenotype (11). These results demonstrate the comprehensive therapeutic effects of the hydrogel through anti-inflammatory, pro-angiogenic, and anti-fibrotic mechanisms, providing a promising multi-targeted treatment approach for myocardial infarction.

### 8.4 Colchicine and ECM

Colchicine can be used to reduce the risk of myocardial infarction and cardiovascular death in patients with atherosclerosis. The specific mechanism is not yet fully clear, but it may alleviate inflammation by interfering with cytoskeleton polymerization and cell adhesion (161). Low-dose colchicine treatment can inhibit the recruitment of macrophages and neutrophils in inflammation (162). Due to its high toxicity, the intake of high-dose colchicine may cause serious side effects, including death and multi-organ failure (163). Local administration of colchicine to the myocardium may increase the colchicine level in the heart while avoiding adverse reactions in other organs (138). Since hydrogels cannot continuously release small molecules, slowly delivering colchicine to the myocardium requires materials with smaller pore sizes. ECM is a natural source of biomaterials. It can be isolated from animal tissues through chemical treatment or freeze-thawing methods (164). ECM retains structural proteins and growth factors in the natural extracellular space (165). Therefore, biomaterials made from ECM can provide space for new cells and regulate various signaling pathways, thereby promoting the wound healing process (166). After cell removal, ECM can be crushed into nanometer or micrometer-sized fragments. Crushed ECM extracted from regenerative zebrafish hearts was injected into mouse MI models, which helped with heart repair and stimulated the proliferation of mouse cardiomyocytes (167). Nanoscale lipid carriers (NLCs) are a relatively new form of lipid carrier, consisting of solid lipids and liquid lipids (168). Drugs can be incorporated into NLCs by pre-mixing the drug powder with melted lipids before producing NLCs, or by the interaction between drug molecules and the surface of NLCs. Due to the crystalline structure of the solid lipid matrix in NLCs, small molecules can be trapped in the core of NLCs and released slowly into the environment (169).

Therefore, some researchers have attempted to achieve slow release of small molecules into the environment by loading appropriate colchicine into the ECM-NLC hydrogel system. After injection into the myocardium, ECM-NLC gels gel within 2 min, remain at the injection site for at least 7 days, and release the load to the heart for more than 2 weeks, enhancing the heart repair ability after myocardial infarction for more than 3 weeks. Additionally, the mechanism of ECM-NLC-colchicine treatment was studied through transcriptome analysis. ECM-NLC-colchicine regulates the early cardiac injury response by weakening inflammation and fibrosis-related signaling pathways (such as the activity of TGF-β, Hippo, and MAPK) (138). Therefore, ECM-NLC hydrogels are a potential method for treating heart damage.

### 8.5 Islet-1 (ISL1)-MSC-derived exos (ISL1-MSC-exos) and Ang-I

MSCs specifically differentiate into cardiomyocytes as a potential way to reverse myocardial injury disease. Previous research has indicated that ISL1 could induce Gcn5 binding to GATA4/Nkx2.5 promoter regions and induce the interactions among Gcn5, HDAC1, G9A and DNMT-1, which upregulated GATA4/Nkx2.5 expression and promoted MSC differentiation into cardiomyocytes (170). The integration of genetically engineered ISL1-MSC-derived exosomes into innovative Ang-I-loaded hydrogels may promote the retention of exos, thereby amplifying their protective effects. Research findings suggest that ISL1-MSC-derived exos can provide therapeutic benefits both *in vitro* and *in vivo*, potentially because of alterations in exosomal content. The Ang-I-loaded gel increased the retention of ISL1-MSC-exos within endothelial cells, enhancing their antiapoptotic, proliferative, and angiogenic properties. Echocardiographic assessments revealed that the Ang-I-loaded gel significantly increased the therapeutic efficacy of ISL1-MSC-exos in the context of AMI. The primary mechanism underlying this effect appears to be the enhanced retention of ISL1-MSC-exos, which bolsters the angiogenic response in the ischemic myocardium. In summary, ISL1-MSC-exos alone have endothelial protective and proangiogenic effects, whereas the Ang-I-loaded gel significantly increased the localization of ISL1-MSC-exos at the ischemic site, thereby promoting endothelial cell survival and angiogenesis and expediting recovery from AMI (139).

### 8.6 dimeric fragment of hepatocyte growth factor (HGFdf) and engineered stromal cell-derived factor 1α (ESA)

Amanda N. Steele et al. encapsulated two protein-engineered cytokines at different stages of the hydrogel to achieve temporal and spatial control of drug delivery and to prolong the release time, namely the HGFdf and ESA (140). The first factor, HGFdf, which has superior stability and expression levels compared to the full-length recombinant HGF (171). The therapeutic effects of HGF have been well established, including anti-apoptotic, anti-fibrotic, promotion of angiogenesis and myocardial generation, etc (172–174). Stromal cell-derived factor 1α (SDF) is a potent chemical inducer of CD34 + stem cells and exhibits significant effects in promoting angiogenesis and wound healing. Therefore, the second cytokine used for the dual-stage release in the hydrogel is the engineered analog of SDF (ESA), which has better stability, functions, simpler synthesis and lower production costs (175–177). Due to the anti-apoptotic activity of HGFdf, it was encapsulated in the aqueous phase to achieve a faster release, in order to minimize myocardial cell apoptosis to the greatest extent. ESA was encapsulated in the nanoparticles stage of the hydrogel to eliminate the time difference between the natural expression of SDF and its receptor CXCR4. In bone marrow cells and myocardial cells, the expression of CXCR4 reached its peak at 96 h after ischemia (176), so ESA was designed to have a slower release rate. Impressively, the hydrogel could release ESA for over 30 days and HGFdf for 28 days *in vitro*. Then, the efficacy of this treatment was evaluated in a mouse myocardial infarction model. Animals treated with the hydrogel encapsulated with ESA and HGFdf showed a significant reduction in scar area, a strong stimulation of angiogenesis in the boundary area, and the preservation of the geometry and function of the left ventricle. All treatment components remained in the cardiac tissue for over 28 days. Finally, given the significant functional and biological benefits observed in the small animal experiments, the HGFdf and ESA loaded hydrogel was successfully delivered to sheep tissues through a catheter-based method, resulting in a reduction in scar area within eight weeks after myocardial infarction. Although no significant changes in functional parameters were observed, this preliminary study will guide methodological improvements and provide valuable insights for future research. For example, the significant reduction in scar area indicates the possibility of achieving therapeutic effects, but such therapeutic effects may require a prolonged period to induce corresponding functional benefits (140). This preliminary study also prompts us to conduct more in-depth investigations into appropriate doses for large animals and longer time points. It may be necessary to conduct longer studies in small animals to understand the appropriate doses. Taken together, these results provide a rationale for the further development of growth factor-based therapies for myocardial repair and regeneration.

## 9 Limitations and translational challenges

Although various synthetic hydrogels perform well, from a translational perspective, they have several drawbacks. In terms of material, natural polymer hydrogels typically exhibit excellent biocompatibility, low cytotoxicity, and similarity to the physiological environment. However, their shortcomings include limited sources, potential immunogenicity, uncontrolled degradation, poor material reproducibility, weak mechanical properties, and difficulties in adjusting the structure and mechanical properties artificially (178).

In terms of application methods, injectable hydrogels usually have relatively low mechanical strength and may trigger an immune response when injected into the heart. Moreover, the application of injectable hydrogels for cardiac treatment aims to reduce the need for open chest surgery. However, the implantation method itself may carry certain risks. A common method is to directly inject the hydrogel through the epicardium into the myocardium. However, this method requires many injections and open chest surgery and is thus highly invasive for the patient, thereby introducing the possibility of surgical complications. Endomyocardial myocardial injection involves the use of a catheter to guide the hydrogel to the site of cardiac injury, and the injection of the hydrogel into the myocardium through the endocardium via the catheter and imaging technology is considered safer and more precise. However, this method requires hydrogels with very specific mechanical properties, limiting the supply of suitable biomaterials. In contrast, coronary artery injection is carried out through the main coronary artery, but there is a risk of coronary artery occlusion or coronary artery re-embolization, thus limiting its clinical application (179). Therefore, a safer method for injecting hydrogels is still needed to promote the development of their clinical application.

From the perspective of clinical efficacy, early studies have demonstrated the safety and potential of injecting alginate hydrogels into coronary arteries. However, the results of clinical trials indicate that the decisive therapeutic benefits and clinical application value still require further research. A phase I clinical trial of Algisyl LVR (NCT00847964) involved three patients with ischemic cardiomyopathy who received an intramyocardial injection of Algisyl LVR during coronary artery bypass grafting. Six months later, cardiac magnetic resonance imaging revealed a reduction in the left ventricular end-diastolic and end-systolic volumes, whereas the stroke volume, left ventricular ejection fraction, and wall thickness increased. Computer model simulation results indicated that the wall stress and stress anisotropy of the patients’ myocardial fibers significantly decreased (180). The phase II clinical AUGMENT-HF trial (NCT01311791) confirmed that implanting Algisyl LVR into the myocardium of patients with heart failure is safe and feasible and can effectively improve patients’ exercise tolerance and heart failure symptoms, as well as enhance cardiac function classification. However, none of the changes in echocardiographic indicators in this study reached statistical significance within the groups (181). IK-5001, developed by Israeli scholars, can penetrate damaged coronary arteries and form gels in a high-calcium environment. However, it cannot pass through normal blood vessels or form nonischemic myocardial gels under normal calcium ion concentrations. A phase I clinical trial (NCT0055753) confirmed that using the coronary artery injection of IK-5001 to treat ST-segment elevation myocardial infarction is safe and feasible and does not affect coronary artery blood flow or myocardial perfusion. After 6 months of follow-up, the patients’ cardiac function remained at the preoperative level, the heart chambers did not further expand, and the level of N-terminal pro-B-type natriuretic peptide gradually decreased (182). Many heart failure therapies require a relatively long time to achieve maximum effects or reverse remodeling; thus, a longer observation period may be needed to fully determine the impact of alginate–water gel treatment on left ventricular remodeling, and further studies are needed to evaluate its therapeutic benefits.

In addition, the transformation of gel therapy from preclinical research to clinical application remains challenging: owing to the biological differences between experimental animals and humans, it is currently unclear whether the injection time, dose, route, and degradation time of the gel can be controlled. Scholars need to prioritize the use of large animal models for clinical prediction verification, adopt standardized reporting standards to improve reproducibility, conduct long-term safety studies to assess biocompatibility, and evaluate sex-specific efficacy to ensure broad applicability.

## 10 Conclusions and future directions

Hydrogels loaded with different drugs, growth factors, drugs, cells, exos and miRNAs provide further possibilities for reducing inflammation levels after MI, regulating immunity, inhibiting fibrosis and promoting cardiac regeneration. It is easy for us to observe that in recent years, water gels carrying two substances have become more common. This, in another aspect, demonstrates the superiority of such gels over those carrying only one therapeutic component. I also prefer this type of water gel that, by carrying different substances, can precisely act on the myocardial injury pathway through different mechanisms, and it may also reduce the production cost of hydrogels. They are promising tools for achieving new treatments for MI, although most of them are still in the preclinical research stage. In clinical applications, owing to the differences in the size of the infarct area, the amount of hydrogel injected in different treatment methods and the time of injection after myocardial infarction need further study. To achieve the best therapeutic effect, personalized injection doses are necessary. With respect to hydrogel materials, future research should focus on developing intelligent gels that interact positively with the microenvironment after myocardial infarction: achieving spatial/temporal controlled release of enzyme-responsive systems; optimizing temperature-responsive systems to achieve optimal gelation; and exploring how different hydrogel materials can degrade while maintaining their mechanical properties. In addition, it is necessary that the fragments of degraded hydrogels should not cause adverse reactions in the body; furthermore, the extensive release of cells, drugs and factors caused by degradation may also affect treatment efficiency or cause side effects. Therefore, a more complete evaluation method like extending the observation period for assessing the efficacy and side effects of hydrogels in the treatment of myocardial infarction is needed.

## Glossary

AMI: acute myocardial infarction
bFGF: basic fibroblast growth factor
VEGF: vascular endothelial growth factor
ECM: extracellular matrix
AS IV: astragalosides IV
TAK1–MAPK: transforming growth factor-β-activated kinase 1 mitogen-activated protein kinase
NOD: nucleotide-binding oligomerization domain
NLRP3: nucleotide-binding oligomerization domain-like receptor thermal protein domain-associated protein 3
TLR4: toll-like receptor 4
NF-κB: nuclear factor-kappa B
ER: endoplasmic reticulum
TXNIP: thioredoxin-interacting protein
IL: interleukin
AMPK: adenosine 5′-monophosphate (AMP)-activated protein kinase
Bcl2: B-cell lymphoma-2 protein
Bax: Bcl-2-associated X protein
Ang: angiopoietin
TPL: *tripterygium wilfordii* lactone
Cx43: connexin 43
SalB: salvianolic acid B
LDL: low-density lipoprotein
ROS: reactive oxygen species
ox-LDL: oxidized low-density lipoprotein
PARP-1: poly ADP-ribose polymerase
PPARγ: peroxisome proliferator-activated receptor γ
HDAC3: histone deacetylase 3
GSK3β: glycogen synthase kinase-3β
FGF: fibroblast growth factor
TGF: transforming growth factor
rhCol III: recombinant human collagen type III
TNF-α: tumor necrosis factor α
HGF: hepatocyte growth factor
Cur: curcumin
IGF: insulin-like growth factor
SF: silk fibroin
NRG: neuregulin
ERK: extracellularly regulated protein kinase
LVEF: left ventricular ejection fraction
MSCs: mesenchymal stem cells
BM-MSCs: bone marrow-derived MSCs
ADSCs: adipose tissue-derived MSCs
hUC-MSCs: human umbilical cord-derived MSCs
CPCs: cardiac progenitor cells
hiPSCs: human induced pluripotent stem cells
ESCs: embryonic stem cells
hiPSC-CMs: hiPSC-derived cardiomyocytes
exos: exosomes
PI3 K: phosphatidylinositol 3-kinase
PGN: peptidoglycan
DCs: dendritic cells
DC-exos: dendritic cell-derived exos
DC-exo gel: DC-exo-loaded alginate hydrogel
miRNA: microRNA
AAV: adeno-associated virus
miNPs: polymer nanoparticles encapsulating miRNAs
MPs: microparticles
ISL1: Islet-1
ISL1-MSC-Exos: ISL1-MSC-derived exos.
